# How should extra‐large Lugol‐unstained lesions of the esophagus be treated? Results from a population‐based cohort study

**DOI:** 10.1002/cam4.6592

**Published:** 2023-09-21

**Authors:** Mengfei Liu, Zifan Qi, Ren Zhou, Chuanhai Guo, Anxiang Liu, Haijun Yang, Fenglei Li, Liping Duan, Lin Shen, Qi Wu, Zhen Liu, Yaqi Pan, Fangfang Liu, Ying Liu, Hong Cai, Zhonghu He, Yang Ke

**Affiliations:** ^1^ State Key Laboratory of Molecular Oncology, Beijing Key Laboratory of Carcinogenesis and Translational Research, Department of Genetics Peking University Cancer Hospital & Institute Beijing China; ^2^ Endoscopy Center Anyang Cancer Hospital Henan Province Anyang China; ^3^ Department of Pathology Anyang Cancer Hospital Henan Province Anyang China; ^4^ Hua County People's Hospital Henan Province China; ^5^ Department of Gastroenterology Peking University Third Hospital Beijing China; ^6^ State Key Laboratory of Holistic Integrative Management of Gastrointestinal Cancers, Beijing Key Laboratory of Carcinogenesis and Translational Research, Department of Gastrointestinal Oncology Peking University Cancer Hospital & Institute Beijing China; ^7^ State Key Laboratory of Holistic Integrative Management of Gastrointestinal Cancers, Beijing Key Laboratory of Carcinogenesis and Translational Research, Endoscopy Center Peking University Cancer Hospital & Institute Beijing China

**Keywords:** endoscopy, ESCC, ESECC, treatment

## Abstract

**Background:**

Current guidelines recommend only severe dysplasia and above (SDA) lesions of the esophageal squamous epithelium for clinical intervention. However, the histopathologic diagnosis derived from tissue biopsies may be subject to underestimation of severity.

**Methods:**

1073 participants from whom biopsies were taken at baseline chromoendoscopic examination in a population‐based screening trial were enrolled in this study. The size of the Lugol‐unstained lesions (LULs) was mainly analyzed. The outcome was defined as SDA lesions either identified at baseline screening, or during follow‐up, collectively referred to as the cumulative risk of SDA. Multivariable logistic regression models were used to evaluate the cumulative risk of SDA.

**Results:**

One hundred and forty‐six SDA cases were identified in the study period. Participants with large LULs had a high cumulative incidence of SDA (cumulative incidence_16–20mm_: 55.88%; cumulative incidence_>20mm_: 76.92%) in the median of 7‐year duration. LULs of large size were significantly associated with a higher cumulative risk of SDA, regardless of the pathologic diagnosis (adjusted OR_16–20mmvs.≤5mm_ = 21.02, 95% CI: 7.56–58.47; adjusted OR_>20mmvs.≤5mm_ = 33.62, 95% CI: 11.79–95.87).

**Conclusions:**

Results from this study suggest physician–patient shared decision‐making regarding clinical treatment or intensive surveillance should be carried out for LULs >20 mm in the esophagus, regardless of the histologic diagnosis. For those with LULs of 16–20 mm, intensive surveillance would also best be considered.

## 1 INTRODUCTION

Esophageal cancer (EC) is a cancer of high prevalence worldwide and was ranked 7th in incidence and 6th in mortality in 2020.^1^ EC ranks 6th and 4th in incidence and mortality respectively when ranked among all cancer types in China.^2^ Over 85% of EC patients are esophageal squamous cell carcinoma (ESCC).^3^ Early‐stage ESCC and its premalignant lesion (esophageal squamous dysplasia) can be identified by endoscopic examination. Endoscopic submucosal dissection (ESD) is regarded as the treatment of preference for early malignant lesions showing severe dysplasia or staged as pTis.^4^, ^5^ The 5‐year survival of early‐stage patients after ESD is about 90%, which is much higher than that of individuals diagnosed at an advanced stage (lower than 30%).^6^ Population‐based investigations have proved that early detection and treatment is crucial for the prevention and control of ESCC.^7^

Lugol's chromoendoscopy (LCE) is the standard method for the detection of early ESCC and precancerous lesions.^6^ Lugol's iodine is applied to visualize the abnormal mucosa, which is unstained under LCE due to the lack of glycogen,^8^ and biopsies are then taken from the Lugol‐unstained lesions (LULs) for histologic examination. Despite the fact that both LCE and magnifying endoscopy with narrow‐band imaging are efficient techniques for identifying and classifying potentially esophageal cancerous lesions, LCE is extensively used in community‐based ESCC screening initiatives in rural areas due to its relatively low cost and simplicity, as well as its high sensitivity for detecting early‐stage precancerous lesions. According to current guidelines, subsequent management of an individual is determined based on the pathologic diagnosis of the biopsy specimen, in which treatment is recommended only for patients with severe dysplasia, carcinoma in situ, or ESCC.^4^

However, results based on histologic analysis of biopsy specimens do not always accurately reflect the malignancy of a given LUL. This is possibly due to the tissue sample not being representative of the lesion and the considerable heterogeneity within a given lesion, especially for large LULs. Studies have shown high discrepancy rates for histopathologic diagnoses biopsied with forceps versus tissue resected from esophageal squamous neoplasms, and 32.1% to 52.9% of patients were underestimated in biopsies.^9^, ^10^, ^11^ As a result, a considerable proportion of early‐stage patients may miss the opportunity for a timely clinical intervention, which would in turn impact long‐term survival, if therapeutic choices are made solely based on biopsy pathology.

To avoid underdiagnosis, an endoscopic re‐examination and re‐biopsy after 3–6 months would usually be recommended for individuals with lesions that are highly suspicious for malignancy under LCE but are not rendered as severe dysplasia and above (SDA) in histologic diagnosis.^4^ Experts on ESCC screening in China have proposed several endoscopic indicators for histologic upgrading (for example, a nodular surface, reddish surface on the lesion, lesion diameter >3 cm),^11^, ^12^ and called for more active treatment for LULs demonstrating those features.^13^ However, these indicators are primarily based on clinical experience and lack robust evidence to support their validity.

In our previous study, we demonstrated that iodine staining features, especially a larger LUL size predicted a higher risk of lesion progression, and we proposed a modified surveillance strategy that includes monitoring non‐dysplastic lesions which are large in size.^14^ However, to evaluate the benefit and necessity of clinical intervention for a given individual, the cumulative risk of malignancy covering both the very time of endoscopic examination and a relatively long period after the examination was more reasonable, for this measurement could avoid outcome misclassification caused by single‐time biopsy and pathologic analysis.

In this study, we evaluated the ability of endoscopic features to determine the applicability of clinical intervention when an LUL was found at LCE. The iodine staining features served as indicators to identify individuals at high cumulative risk of having malignant lesions, based on a large‐scale population‐based ESCC screening cohort in a high‐risk region in China with up to 9‐year follow‐up. This study aims to provide evidence for refining the criteria regarding the clinical intervention of esophageal squamous lesions.

## 2 METHODS

### 2.1 Study subjects

Participants in this study were selected from the Endoscopic Screening for Esophageal Cancer in China (ESECC) randomized controlled trial (Clinical trial: NCT01688908) initiated in Hua county, Henan province, which was a high‐risk area for EC in China in January 2012.^15^ 668 villages were randomly selected from the 846 villages in this county and were equally allocated into the screening arm or the control arm of the study using a blocked randomization procedure. Standard upper gastrointestinal endoscopic examination was performed in the screening arm, while no screening was offered to participants in the control arm. The inclusion criteria for the ESECC trial can be found elsewhere.^15^

For the current study, we enrolled ESECC participants in the screening arm who had LULs which were observed at baseline endoscopic screening and from whom biopsy samples were collected. Participants were excluded if their baseline endoscopic images were not clear enough for retrospective review of LUL features.

### 2.2 Questionnaire investigation

A computer‐aided one‐on‐one questionnaire was used to collect information on potential ESCC risk factors from each participant prior to endoscopy at the baseline investigation.^16^ Potential ESCC risk factors included in the questionnaire were age, gender, body mass index (BMI), ESCC family history, socioeconomic status, lifestyle information, and ESCC‐related symptoms.^17^

### 2.3 Endoscopic examination and endoscopic image documentation

Participants enrolled in this study were screened using standard upper gastrointestinal endoscopy with 1.2% Lugol's iodine solution at baseline. The entire length of the esophagus was visually examined under white light and then with LCE. Endoscopic images were routinely captured along the esophagus every 5 cm. Biopsies were taken from areas with abnormalities under white light or LCE, and extra images were captured for each LUL prior to biopsy for retrospective review.

### 2.4 Pathologic diagnosis

Biopsy specimens were fixed in 10% formaldehyde, embedded in paraffin, sectioned at 5 μm, and stained with hematoxylin and eosin. The specimen sections were read by two experienced pathologists from Anyang Cancer Hospital who were blinded to endoscopic findings. Discrepancies were resolved by consultation.^15^ The specimens were graded as follows in the histologic diagnoses: no dysplasia (including normal, acanthosis, esophagitis, and basal cell hyperplasia), mild dysplasia, moderate dysplasia, severe dysplasia, carcinoma in situ, and squamous cell carcinoma. For participants with more than one LULs biopsied, the one with the highest pathologic grade was used for this study.

### 2.5 Endoscopic image review

Baseline endoscopic images of the study participants were retrospectively reviewed by well‐trained researchers who did not know the histologic diagnoses. Discrepancies were resolved by discussion. Based on review of the literature and our previous study results, we assessed 6 endoscopic features in this study, including the size of LULs, mosaic staining, irregularity, sharpness of the border, dark staining border, and numbers of LUL in the entire esophagus.^14^, ^18^, ^19^ The features were extracted from the LUL with the highest grade pathologic diagnosis if more than one LUL were found in the esophagus. Details regarding the measurements of endoscopic features of LULs are presented in the supplementary material (supplementary material: definition and Figure S1). Among these, features of LUL of interest were LUL size (defined as the diameter or the length, whichever was smaller), and LUL size was categorized into 4 groups, namely small LUL (≤5 mm), medium LUL (6–15 mm), large LUL (16–20 mm), and extra‐large LUL (>20 mm).

### 2.6 Follow‐up and outcome ascertainment

The outcome of this study was defined as SDA cases, including severe dysplasia, carcinoma in situ, and ESCC identified either at baseline screening or in the follow‐up of the ESECC trial. The cumulative incidence was calculated as the total number of SDA cases diagnosed at baseline or during follow‐up divided by number of participants.

Prevalent cases of SDA were defined as those identified at the baseline screening or within 1 year after the baseline screening. For participants with no prevalent SDA, incident SDA cases were determined by three approaches in the follow‐up of the ESECC trial, which exhibited a sensitivity of >95% and a specificity of nearly 100% in identifying cancer cases according to our previous assessment.^20^, ^21^, ^22^ The first approach was active annual door‐to‐door interviews, completed by village doctors of the included villages. In the second approach, passive follow‐up was conducted annually by linkage using personal ID with the claims data of the New Rural Cooperative Medical Scheme. The latter had a coverage of nearly 100% in the study area.^23^ Third, we invited all the participants enrolled in this study to undergo a surveillance LCE examination in the period from May 2017 to November 2018,^14^ and included SDA cases detected at the exact location which was documented in the initial screening. The administrative censoring date of follow‐up in this study was set as November 15, 2020.

### 2.7 Statistical analysis

As LUL size was a key determinant of the probability of malignancy, Kaplan–Meier curves were plotted by different groups of LUL size, visualizing the cumulative probability of having SDA among all participants. Log‐rank testing was used to compare differences between groups, and the multiple testing for post‐hoc pairwise comparison was adjusted using the Benjamini and Hochberg method.

Logistic regression models were applied to evaluate the association of the potential risk factors with the cumulative risk of SDA in the study period. We selected epidemiological variables that were generally accepted as risk factors for esophageal malignant lesions including demographic information (age, gender, family history of ESCC, BMI) and behavioral information (alcohol consumption, cigarette smoking, eating rapidly, and taking leftovers)^15^, ^24^; and endoscopic variables including features of LUL (size, mosaic staining, irregularity, sharpness of the border, dark staining border, and numbers of LUL), and the pathologic diagnosis at baseline. All covariates were adjusted in a multivariable logistic regression model to evaluate the association between LUL size and cumulative risk of SDA.

Analysis stratified by pathologic diagnosis was also performed in the subset of participants with no SDA, no dysplasia, and mild dysplasia and above at baseline separately, to evaluate the effect of potential risk factors in different stages of the diseases. We also calculated the detection rate of SDA at baseline among all participants, as well as the cumulative incidence of SDA among the participants with no SDA at baseline.

Statistical analysis was performed using Stata (version 16.1, StataCorp) and R version 4.2.1 (R Foundation for Statistical Computing). All tests were two‐sided and had a significance level of 0.05.

### 2.8 Ethics statement

Research protocols were approved by the Institutional Review Board of the Peking University School of Oncology, Beijing, China. All participants provided written informed consent.

## 3 RESULTS

**FIGURE 1:**
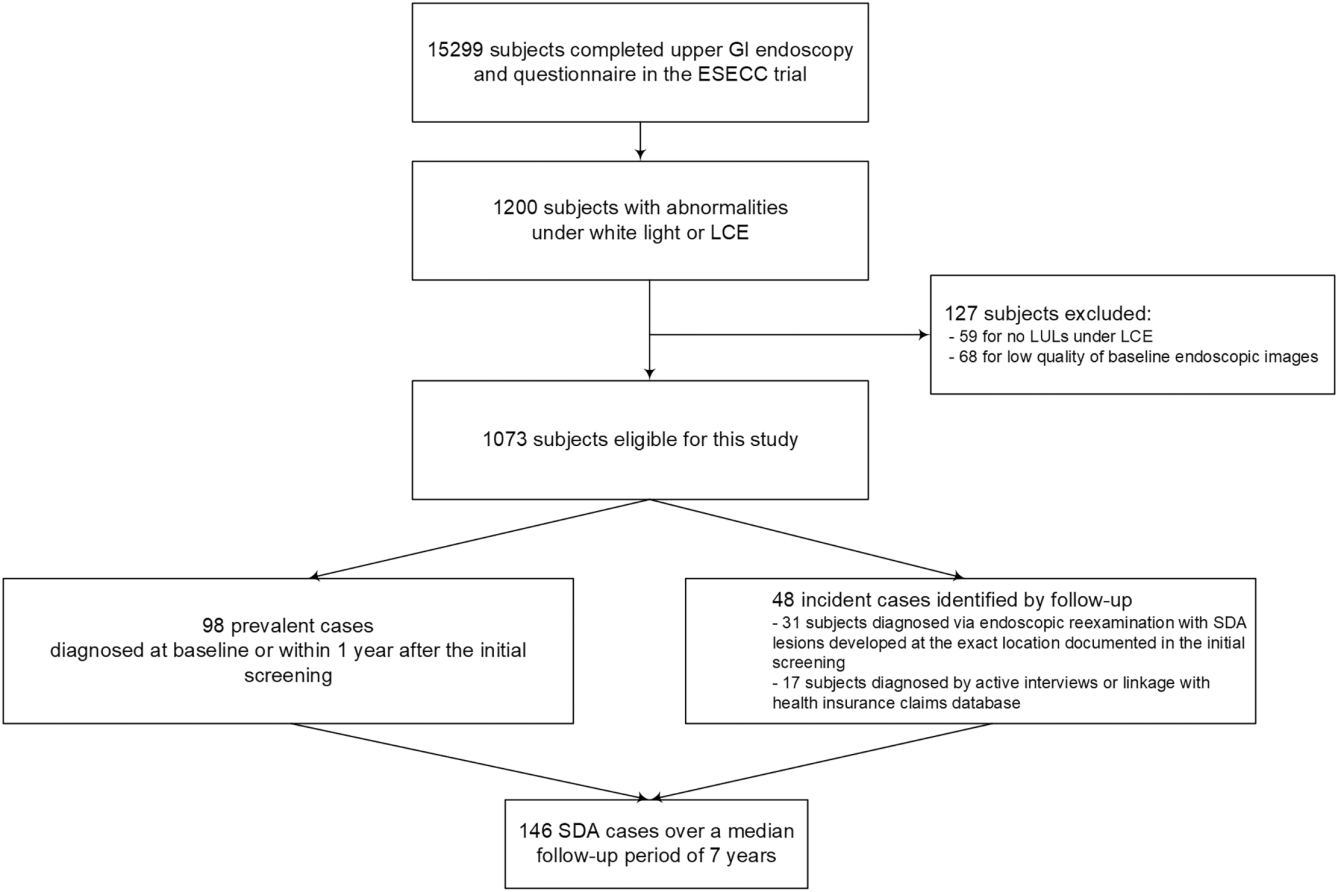
Flowchart illustrating the inclusion of participants and identification of SDA cases in the current study. ESECC, Endoscopic Screening for Esophageal Cancer in China; GI, gastrointestinal; LCE, Lugol's chromoendoscopy; LUL, Lugol‐unstained lesion; SDA, severe dysplasia and above.

A total of 1073 participants who met inclusion criteria were analyzed in this study. In the course of follow‐up of a median of 7.02‐years (interquartile range: 5.57–8.45 years), 146 SDA cases were identified, including 98 prevalent cases and 48 incident cases (Figure 1). Participants of older age, BMI ≤22 kg/m^2^, with LULs with a sharp border, irregularity, mosaic staining under LCE, a baseline histologic diagnosis of mild dysplasia and above, LULs of larger size, and subjects with multiple LULs had a significantly higher cumulative risk of SDA (Table 1). The cumulative incidence of having SDA in participants with no dysplasia, mild dysplasia and above were 2.72% (95% CI: 1.69%–4.13%) and 41.53% (95% CI: 35.90%–47.32%) in a median of 7 years, respectively. The cumulative incidence of SDA for participants with LUL size of ≤5 mm, 6–15 mm, 16–20 mm, and > 20 mm were 3.90% (95% CI: 2.52%–5.75%), 18.96% (95% CI: 15.17%–23.24%), 55.88% (95% CI: 37.89%–72.81%), and 76.92% (95% CI: 60.67%–88.87%), respectively, in a median of 7 years (Table 1).

**TABLE 1:** Characteristics of selected demographic factors, behavioral variables and features of LULs at baseline endoscopy in 1073 participants in the ESECC trial in rural Hua County, China, 2012–2020.

Variables	No. of non‐SDA cases/No. of SDA cases	Crude ORs^a^		Cumulative incidence of SDA & 95% CI (per 100 persons)^b^
		OR (95% CI)	*p*‐Value	
Age (years)	–	1.12 (1.07–1.16)	<0.001	–
Gender				
Female	435/74	Ref		14.54 (11.59–17.90)
Male	492/72	0.86 (0.61–1.22)	0.398	12.77 (10.12–15.80)
BMI				
> 22 kg/m^2^	736/102	Ref		12.17 (10.03–14.58)
≤ 22 kg/m^2^	191/44	1.66 (1.13–2.45)	0.010	18.72 (13.95–24.31)
Family history of ESCC				
No	800/118	Ref		12.85 (10.76–15.19)
Yes	127/28	1.50 (0.95–2.35)	0.082	18.06 (12.35–25.04)
Alcohol consumption				
No	664/116	Ref		14.87 (12.45–17.57)
Yes	263/30	0.65 (0.43–1.00)	0.050	10.24 (7.02–14.29)
Cigarette smoking				
No	544/94	Ref		14.73 (12.07–17.72)
Yes	383/52	0.79 (0.55–1.13)	0.193	11.95 (9.06–15.38)
Eating rapidly				
No	142/21	Ref		12.88 (8.16–19.02)
Yes	785/125	1.08 (0.66–1.77)	0.770	13.74 (11.57–16.15)
Ingestion of leftovers				
≤ 1 time per week	550/82	Ref		12.97 (10.45–15.85)
> 1 time per week	377/64	1.14 (0.80–1.62)	0.470	14.51 (11.36–18.15)
Sharp border				
No	380/44	Ref		10.38 (7.64–13.68)
Yes	547/102	1.61 (1.11–2.35)	0.013	15.72 (13.00–18.75)
Irregularity				
No	222/10	Ref		4.31 (2.09–7.78)
Yes	705/136	4.28 (2.21–8.28)	<0.001	16.17 (13.75–18.84)
Mosaic staining				
No	338/25	Ref		6.89 (4.51–10.00)
Yes	589/121	2.78 (1.77–4.36)	<0.001	17.04 (14.35–20.01)
Dark staining border				
No	623/98	Ref		13.59 (11.17–16.31)
Yes	304/48	1.00 (0.69–1.46)	0.984	13.64 (10.23–17.67)
Numbers of LULs				
1	628/75	Ref		10.67 (8.48–13.19)
≥ 2	299/71	1.99 (1.40–2.83)	<0.001	19.19 (15.30–23.58)
Pathology at baseline				
No dysplasia	751/21	Ref		2.72 (1.69–4.13)
Mild dysplasia and above	176/125	25.40 (15.55–41.48)	<0.001	41.53 (35.90–47.32)
Size of LULs^c^				
Small (≤5 mm)	591/24	Ref		3.90 (2.52–5.75)
Medium (6–15 mm)	312/73	5.76 (3.56–9.32)	<0.001	18.96 (15.17–23.24)
Large (16–20 mm)	15/19	31.19 (14.15–68.76)	<0.001	55.88 (37.89–72.81)
Extra‐large (>20 mm)	9/30	82.08 (35.11–191.93)	<0.001	76.92 (60.67–88.87)

Abbreviations: BMI, body mass index; CI, confidence interval; ESCC, esophageal squamous cell carcinoma; ESECC, Endoscopic Screening for Esophageal Cancer in China; LUL, Lugol‐unstained lesion; OR, odds ratio; SDA, severe dysplasia and above.

^a^ The crude ORs were derived from univariate logistic regression models.

^b^ The cumulative incidences of SDA of a median of 7 years were calculated as the total of outcome events identified at baseline screening or via follow‐up, divided by the number of participants at enrollment in each subgroup. 95% CI was derived from the binomial distribution.

^c^ Size was defined as the diameter or length, whichever was smaller, of a given LUL.

**FIGURE 2:**
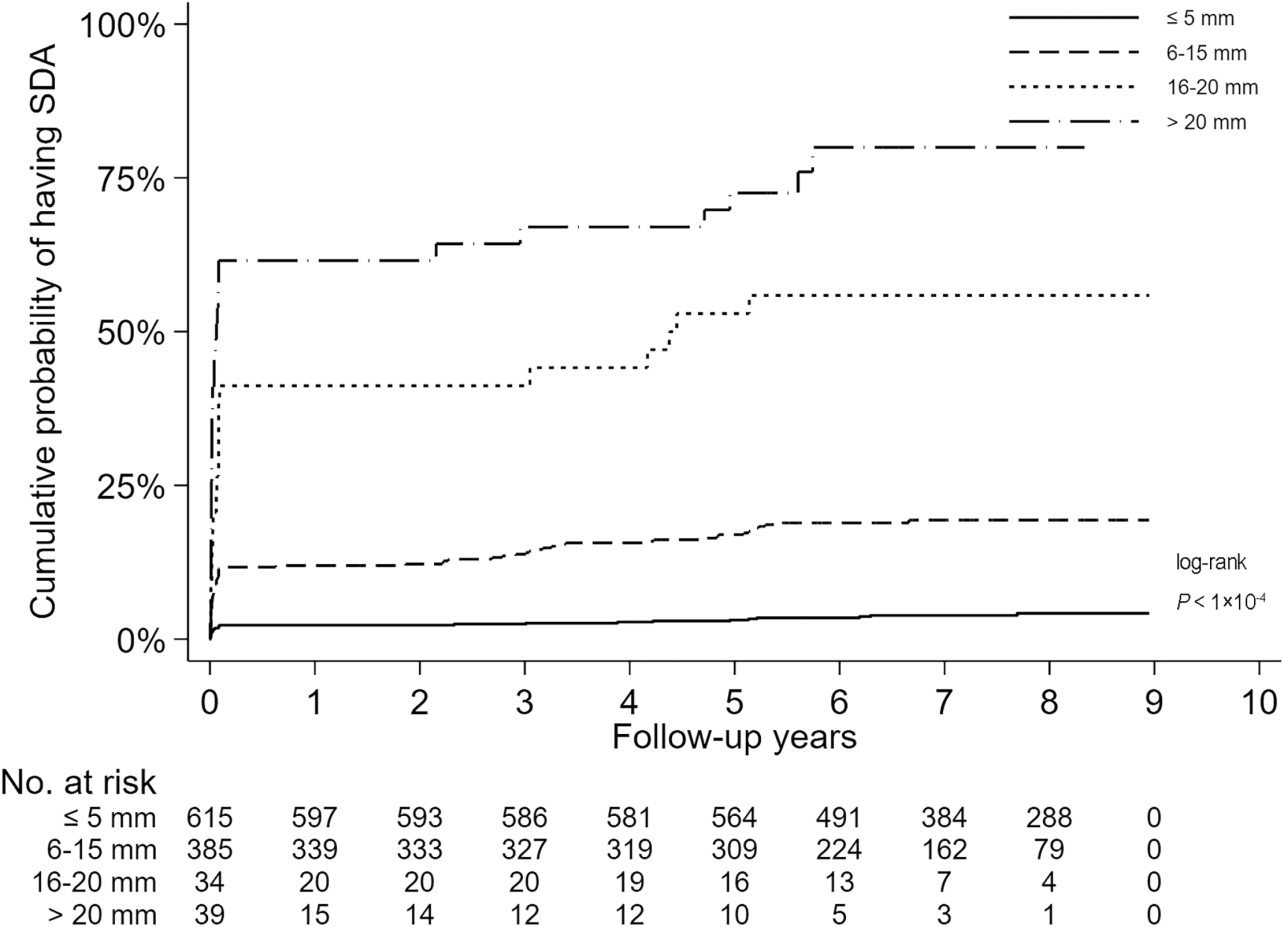
Cumulative probability of SDA for four groups of subjects categorized by LUL size among all participants (*N* = 1073). LUL, Lugol‐unstained lesion; SDA, severe dysplasia and above.

The cumulative probability of SDA increases in a manner directly proportional to the increase in the size of the LULs (Figure 2). The participants with LUL > 15 mm had a remarkably higher cumulative probability of SDA than those with LUL ≤15 mm during a follow‐up period of up to 9 years.

As shown in Table 2, larger LUL size (adjusted OR_6–15mmvs.≤5mm_ = 3.63, 95% CI: 2.05–6.42; adjusted OR_16–20mmvs.≤5mm_ = 21.02, 95% CI: 7.56–58.47; adjusted OR_>20mmvs.≤5mm_ = 33.62, 95% CI: 11.79–95.87) was associated with a significantly higher cumulative risk of SDA after adjusting for epidemiological variables, endoscopic variables of iodine staining features, and pathology at baseline. Large LUL size was also positively associated with higher cumulative risk of SDA among non‐SDA participants (Table 2), as well as those with or without a dysplastic lesion detected at baseline (Table S1). When LUL size was categorized into 5 groups by an increase every 5 mm, the results were consistent (Figure S2; Table S2).

**TABLE 2:** Multivariable analysis of risk factors for SDA among all participants and non‐SDA participants in rural Hua County, China, 2012–2020.

Variables	All (*N* = 1073)		In Non‐SDA (*N* = 975)	
	Adjusted ORs^a^		Adjusted HRs^b^	
	OR (95% CI)	*p*‐Value	HR (95% CI)	*p*‐Value
Age (years)	1.05 (1.00–1.11)	0.042	1.08 (1.01–1.15)	0.029
Gender				
Female	Ref		Ref	
Male	1.62 (0.81–3.21)	0.170	1.07 (0.49–2.36)	0.863
BMI				
> 22 kg/m^2^	Ref		Ref	
≤ 22 kg/m^2^	2.12 (1.24–3.65)	0.006	2.28 (1.21–4.29)	0.011
Family history of ESCC				
No	Ref		Ref	
Yes	1.12 (0.61–2.06)	0.717	0.89 (0.39–2.01)	0.781
Alcohol consumption				
No	Ref		Ref	
Yes	0.82 (0.41–1.62)	0.564	0.79 (0.31–2.02)	0.627
Cigarette smoking				
No	Ref		Ref	
Yes	0.60 (0.29–1.24)	0.168	0.77 (0.32–1.84)	0.555
Eating rapidly				
No	Ref		Ref	
Yes	0.79 (0.42–1.47)	0.455	0.55 (0.28–1.09)	0.088
Ingestion of leftovers				
≤ 1 time per week	Ref		Ref	
> 1 time per week	0.98 (0.60–1.58)	0.923	0.73 (0.38–1.40)	0.346
Sharp border				
No	Ref		Ref	
Yes	1.93 (1.12–3.31)	0.017	1.55 (0.78–3.10)	0.214
Irregularity				
No	Ref		Ref	
Yes	2.01 (0.88–4.61)	0.100	1.73 (0.57–5.20)	0.330
Mosaic staining				
No	Ref		Ref	
Yes	0.69 (0.37–1.30)	0.252	1.37 (0.53–3.56)	0.517
Dark staining border				
No	Ref		Ref	
Yes	0.86 (0.51–1.44)	0.571	1.64 (0.89–3.02)	0.115
Numbers of LULs				
1	Ref		Ref	
≥ 2	1.17 (0.72–1.90)	0.527	1.26 (0.68–2.32)	0.463
Pathology at baseline				
No dysplasia	Ref		Ref	
Mild dysplasia and above	15.42 (8.94–26.60)	<0.001	2.91 (1.58–5.37)	<0.001
Size of LULs^c^				
Small (≤5 mm)	Ref		Ref	
Medium (6–15 mm)	3.63 (2.05–6.42)	<0.001	3.19 (1.46–6.99)	0.004
Large (16–20 mm)	21.02 (7.56–58.47)	<0.001	9.26 (2.86–29.95)	<0.001
Extra‐large (>20 mm)	33.62 (11.79–95.87)	<0.001	11.90 (3.80–37.26)	<0.001

Abbreviations: BMI, body mass index; CI, confidence interval; ESCC, esophageal squamous cell carcinoma; HR, hazard ratio; LUL, Lugol‐unstained lesion; OR, odds ratio; SDA, severe dysplasia and above.

^a^ The adjusted ORs were derived from the multivariable logistic regression model comprising age, gender, BMI, ESCC family history, alcohol consumption, cigarette smoking, eating rapidly, ingestion of leftovers, sharp border of LUL, irregularity, mosaic staining, dark staining border, number of LULs, pathology at baseline and size of LULs in full data set.

^b^ The adjusted HRs were derived from the multivariable Cox regression model comprising age, gender, BMI, ESCC family history, alcohol consumption, cigarette smoking, eating rapidly, ingestion of leftovers, sharp border of LUL, irregularity, mosaic staining, dark staining border, number of LULs, pathology at baseline and size of LULs among non‐SDA participants at baseline.

^c^ Size was defined as the diameter or length, whichever was smaller, of a given LUL.

## 4 DISCUSSION

According to current clinical practice guidelines, immediate treatment is recommended only if an esophageal squamous lesion is graded as SDA by histologic analysis.^4^, ^5^ However, it is widely accepted that the grade of a given lesion may be underestimated in histologic examination secondary to inadequate representativeness of tissue samples collected, resulting in a delay of treatment for some patients with early malignant lesions. Although re‐biopsy and diagnosis within 3–6 months would typically be recommended for lesions that are highly suspicious for malignancy but have not been confirmed as such via histologic diagnosis, judgment by endoscopists regarding LUL under LCE has had little impact on the decision regarding clinical intervention thus far.

In our previous study, we proposed that it would be better to subject individuals with non‐SDA LULs >10 mm in size to endoscopic surveillance every year,^14^ mainly due to their progression rate to SDA. In this study, we further demonstrated that LUL size is also a crucial indicator for identification of persons at high‐risk for SDA at the same time during endoscopic examination and subsequently, irrespective of the results of histologic analysis. This finding for the first time provides evidence based on a large‐scale population‐based screening cohort regarding the importance of visual features of LULs observed under LCE and demonstrates the significance of endoscopists' judgment in determination of the therapeutic strategy for early malignant lesions in the esophagus.

With the advancement of medical technology, clinical intervention of ESCC includes various clinical treatment methods, such as endoscopic therapy and minimally invasive esophagectomy.^4^, ^5^, ^6^ Our study highlights the potential risks of large LULs and calls for cautious management and intervention to prevent malignancy. These findings could provide valuable insights for refining ESCC clinical intervention guidelines in the future.

**FIGURE 3:**
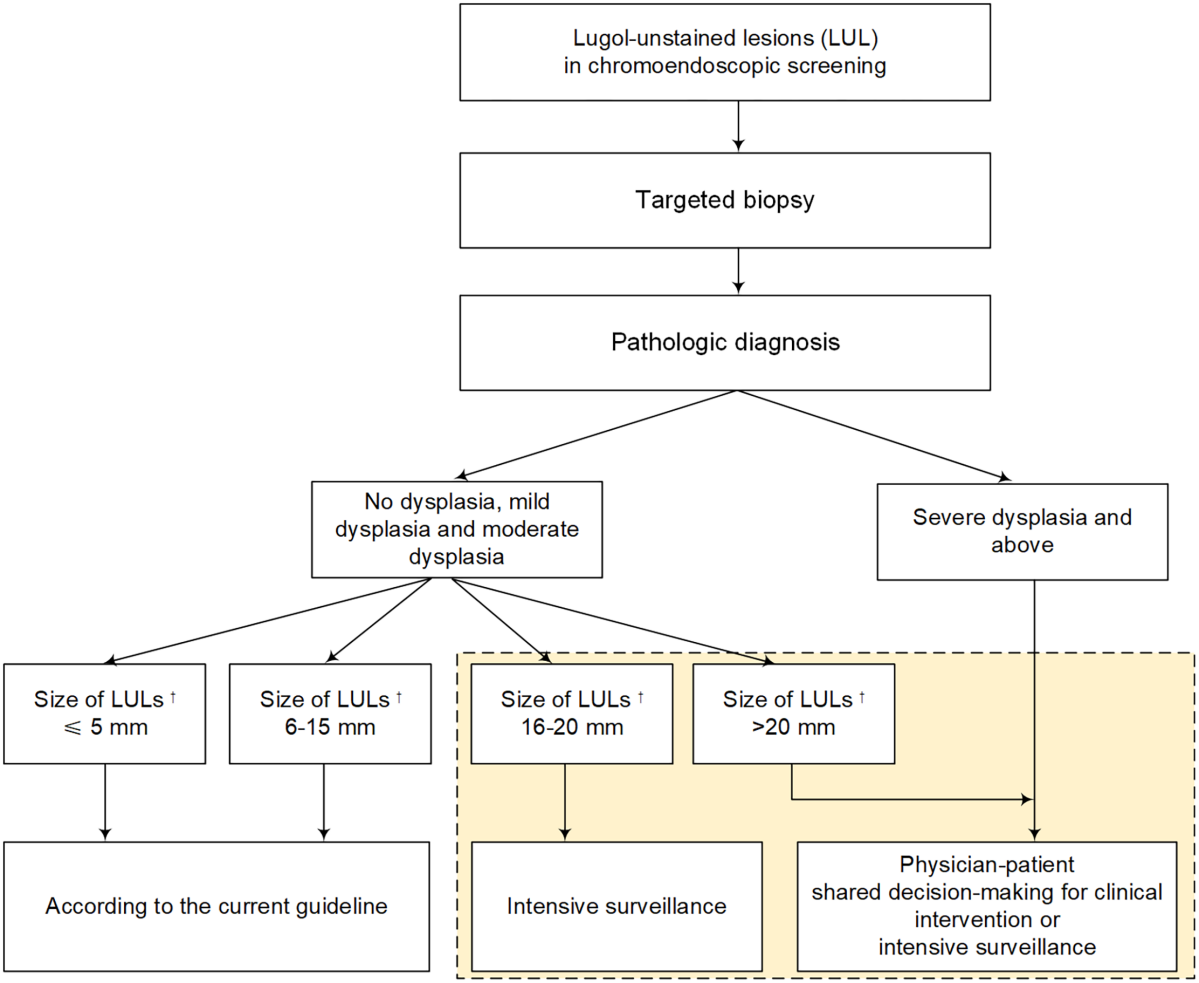
A suggested flowchart of management of LULs depending on LUL size and pathologic diagnosis. LUL, Lugol‐unstained lesion. ^†^: Size was defined as the diameter or length of a given LUL, whichever was smaller.

We suggest that clinicians engage these individuals in shared decision‐making regarding clinical intervention or intensive surveillance for participants with LUL size >20 mm, without waiting for a “final judgment” of SDA, and thereby facilitate timely intervention of early malignant lesions or lesions that are highly likely to progress to malignancy in the future (Figure 3). The reasons for this recommendation were as follows: (1) The cumulative incidence of SDA was as high as 76.92% within a median of 7 years, and 61.54% of the individuals with extra‐large LULs would be identified as prevalent cases by targeted biopsy and histologic analysis (Table S3). (2) Among those not graded as SDA at baseline screening, 40.00% would eventually progress to SDA during a median of 7 years (Table S3), which was much higher than the progression rate of 7.44% reported for people with mild‐to‐moderate dysplasia based on a 7‐year multicentric community‐based surveillance cohort.^25^

For individuals with LUL size of 16–20 mm, the cumulative incidence of SDA was 55.88%, with a prevalence rate of 41.18% and the proportion of non‐SDA participants developing incident SDA during a median of 7 years being 25.00% (Table S3). As a result, intensive endoscopic surveillance should be recommended for these individuals. (Figure 3).

It should be noted that the recommendations we have proposed above based on the LUL size are primarily for use by endoscopists at the time endoscopic examinations are being performed. The inclusion of this macro‐morphologic indicator has the potential to mitigate the impact of pathologic underestimation. In addition, endoscopists may engage the patients with clinical intervention recommendations or close surveillance strategy after the endoscopic examination regardless of the pathologic diagnosis. Thus, a novel modality for early detection and treatment of ESCC may result, in which endoscopists may play an essential role in determining the strategy for clinical intervention or surveillance for individuals with large LULs.

This study has two strengths. First, it was based on a real‐world large‐scale population‐based ESCC screening cohort, which ensured the ideal representativeness of the main conclusions. Second, we included both the SDA cases detected at baseline screening and those ascertained in up to 9‐years follow‐up, thereby avoiding misclassification of the outcome at initial screening, for example, avoiding the underdiagnosis resulting from inadequate representativeness of biopsy sampling.

Limitations of the current study should also be noted. First, this was a single‐center study, and validation with multicenter studies is warranted. Second, the features of LULs were extracted from the still images retrospectively, which may not be as informative as real‐time images during endoscopic examination. Since the observation of the pink color sign requires real‐time assessment during endoscopy and these data were unavailable for this study, which could potentially result in “loss of protection” for the identification and timely intervention of small lesions with a high likelihood of malignancy.

In summary, LUL size (defined as the diameter or the length, whichever was smaller) under LCE is a significant indicator that should be of value to endoscopists in determining the risk of SDA at the time of endoscopic examination and in the future. Our results provide evidence for the refinement of current clinical practices regarding the intervention of early malignant lesions in the esophagus. Patients with extra‐large LUL (>20 mm) would be likely to benefit from physician–patient shared decision‐making of clinical intervention or intensive surveillance regardless of the histologic diagnosis. More concern should also be attached to individuals with large LULs (16–20 mm) regarding their elevated risk of cancer.

## AUTHOR CONTRIBUTIONS

**Mengfei Liu:** Conceptualization (equal); formal analysis (equal); funding acquisition (equal); investigation (equal); writing – original draft (equal). **Zifan Qi:** Formal analysis (equal); investigation (equal); writing – original draft (equal). **Ren Zhou:** Investigation (equal). **Chuanhai Guo:** Investigation (equal). **Anxiang Liu:** Investigation (equal). **Haijun Yang:** Investigation (equal). **Fenglei Li:** Investigation (equal). **Liping Duan:** Investigation (equal). **Lin Shen:** Investigation (equal). **Qi Wu:** Investigation (equal). **Zhen Liu:** Investigation (equal). **Yaqi Pan:** Investigation (equal). **Fangfang Liu:** Investigation (equal). **Ying Liu:** Investigation (equal). **Hong Cai:** Investigation (equal). **Zhonghu He:** Conceptualization (equal); formal analysis (equal); funding acquisition (equal); investigation (equal); supervision (equal); writing – original draft (equal); writing – review and editing (equal). **Yang Ke:** Conceptualization (equal); formal analysis (equal); investigation (equal); supervision (equal); writing – original draft (equal); writing – review and editing (equal).

## CONFLICT OF INTEREST STATEMENT

There are no conflicts of interest with regard to the publication of this study.

## CLINICAL TRIAL REGISTRATION

Endoscopic Screening for Esophageal Cancer in China (ESECC) randomized controlled trial (Clinical trial: NCT01688908).

## Supporting information


Data S1:

## Data Availability

The data are not available for public access due to the privacy concerns of the participants.
