# Supplementary material for: How should extra‐large Lugol‐unstained lesions of the esophagus be treated? Results from a population‐based cohort study
Source: Cancer Med. 2023 Sep 21;12(19):20129–39. doi: 10.1002/cam4.6592 (PMC10587922; doi:10.1002/cam4.6592)
Supplement: Supplementary file 1 — Data S1: [file CAM4-12-20129-s001.docx]

**Supplementary Material**

**Information Regarding Definitions:**

1. **Age**

The age of each subjects at baseline screening was calculated as: *Round* ( $\frac{Date(interview)-Date(birthday)}{365.25}$ ).

1. **Body mass index (BMI)**

BMI was calculated as body weight in kilograms divided by the square of body height in meters (kg/m^2^). Subjects were classified into 2 groups as BMI > 22 (coded as 0) and BMI ≤ 22 (coded as 1).

1. **Family History of Esophageal Squamous Cell Carcinoma (ESCC)**

Family history of ESCC was defined as subjects having ESCC cases in the immediate family and relatives within 3 generations. Subjects were classified into 2 groups as no family history of ESCC (coded as 0) and having family history of ESCC (coded as 1).

1. **Alcohol consumption**

Alcohol consumption was defined as having a current or previous history of drinking Chinese liquor (containing > 40% alcohol). Subjects were classified into 2 groups as no alcohol consumption (coded as 0) and having alcohol consumption (coded as 1).

1. **Cigarette smoking**

Cigarette smoking was defined as having a current or previous history of smoking. Subjects in the current study were classified into 2 groups as no cigarette smoking (coded as 0) and having cigarette smoking (coded as 1).

1. **Eating rapidly**

There are 3 options for this question. Subjects selecting (a) slow were coded as 0, and those selecting (b) sometimes fast, and (c) always fast engaged in eating rapidly were coded as 1.

1. **Ingestion of leftovers**

Subjects who had ingestion of leftovers ≤ 1 time per week were coded as 0 and those had ingestion of leftovers > 1 time per week were coded as 1.

1. **Features of endoscopic images at baseline**

**①** Size was defined as the diameter or length, whichever was smaller, of a given Lugol-unstained lesion (LUL).

**②** Mosaic staining, known as uniformity, was defined as positive if the color of a LUL was not uniform, or showing a “mosaic staining pattern” of multiple interconnected unstained areas.

**③** Irregularity was defined as positive if an irregular border accounted for at least one-half of the circumference of the LUL.

**④** Sharp LUL border was defined as positive if a distinct (sharp) border accounted for at least one-half of the circumference of the LUL.

**⑤** A dark staining border was defined as positive if dark staining border accounted for at least one-half of the circumference of the LUL.

**⑥** The number of LULs was counted when satisfied the following conditions: 1) the size of LUL was > 5 mm; 2) its border was located > 2 cm away from the border of the nearest LUL.


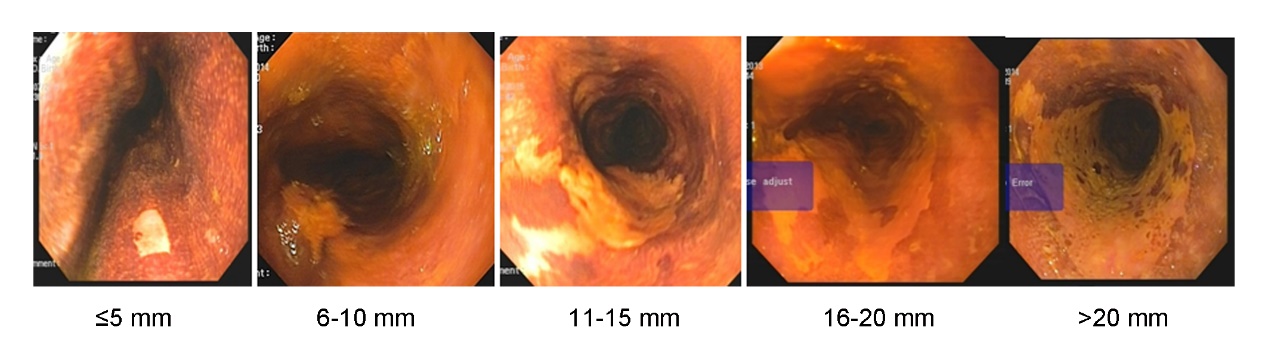


**Supplementary Figure 1.** **Typical endoscopic pictures showing the size of a given LUL.**

Abbreviation: LUL, Lugol-unstained lesion.


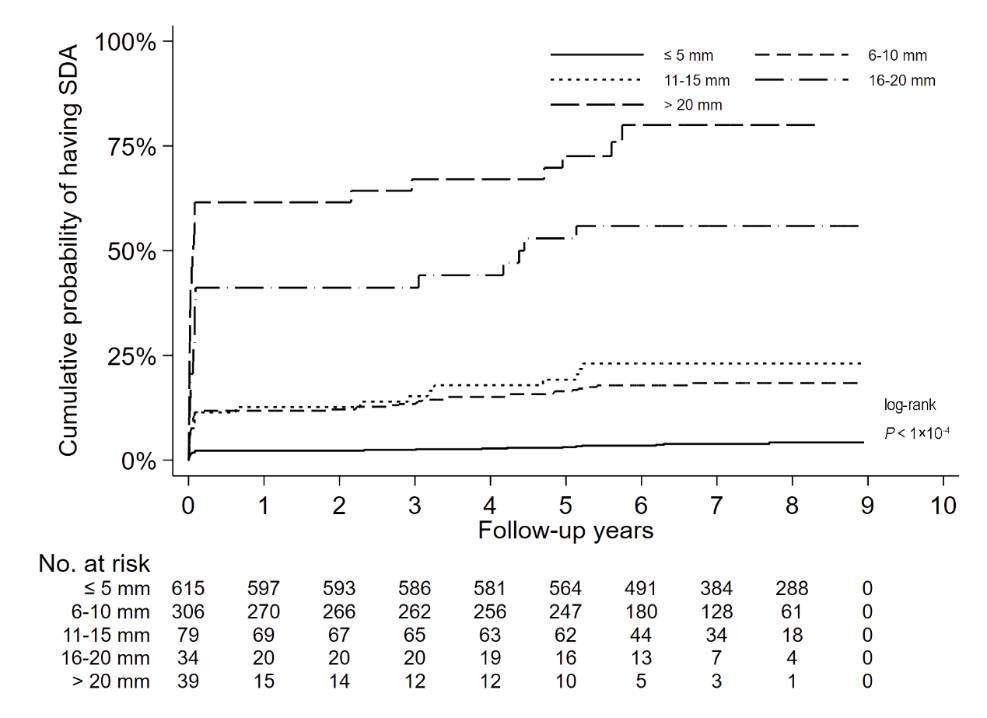


**Supplementary Figure 2. Cumulative probability of having SDA for 5 groups of subjects categorized by LUL size among all participants (N=1073).**

Abbreviations: LUL, Lugol-unstained lesion; SDA, severe dysplasia and above

| **Supplementary Table 1. Multivariable logistic analysis of risk factors for SDA stratified by pathology at baseline generated from 1073 participants in the ESECC trial** | | | | | |
| --- | --- | --- | --- | --- | --- |
|  |  |  |  |  |  |
| Variables | In no dysplasia (N=772) | |  | In mild dysplasia and above (N=301) | |
|  | Adjusted ORs ^†^ | |  | Adjusted ORs ^‡^ | |
|  | OR (95% CI) | *P* value |  | OR (95% CI) | *P* value |
| Size of LULs ^§^ |  |  |  |  |  |
| Small (≤5 mm) | Ref |  |  | Ref |  |
| Medium (6-15 mm) | 13.07 (2.58-66.11) | 0.002 |  | 2.32 (1.23-4.39) | 0.010 |
| Large (16-20 mm) | 153.91 (18.21-1300.73) | <0.001 |  | 8.60 (2.72-27.21) | <0.001 |
| Extra-large (>20 mm) | 233.19 (16.79-3239.42) | <0.001 |  | 17.56 (5.52-55.81) | <0.001 |
| Abbreviations: CI, confidence interval; ESECC, Endoscopic Screening for Esophageal Cancer in China; LUL, Lugol-unstained lesion; OR, odds ratio; SDA, severe dysplasia and above. ^†^ The adjusted ORs were derived from the multivariable logistic regression adjusted by age, gender, body mass index, family history of esophageal squamous cell carcinoma, alcohol consumption, cigarette smoking, eating rapidly, ingestion of leftovers, sharp border of LUL, irregularity, mosaic staining, dark staining border and number of LULs among participants with no dysplasia lesion at baseline.  ^‡^ The adjusted ORs were derived from the multivariable logistic regression adjusted by age, gender, body mass index, family history of esophageal squamous cell carcinoma, alcohol consumption, cigarette smoking, eating rapidly, ingestion of leftovers, sharp border of LUL, irregularity, mosaic staining, dark staining border and number of LULs among participants diagnosed as mild dysplasia and above at baseline.  ^§^ Size was defined as the diameter or length, whichever was smaller, of a given LUL. | | | | | |
|  |  |  |  |  |  |
|  |  |  |  |  |  |

| **Supplementary Table 2. The crude ORs, adjusted ORs, and cumulative incidence of SDA in 5 categories of LUL size from 1073 participants in this study** | | | | | | | |
| --- | --- | --- | --- | --- | --- | --- | --- |
| Variables | No. of non-SDA cases/  No. of SDA cases | Crude ORs ^†^ | |  | Adjusted ORs ^‡^ | | Cumulative incidence of SDA & 95% CI  (per 100 persons) ^§^ |
|  |  | OR (95% CI) | *P* value |  | OR (95% CI) | *P* value |  |
| Size of LULs ^¶^ |  |  |  |  |  |  |  |
| ≤5 mm | 591/24 | Ref |  |  | Ref |  | 3.90 (2.52-5.75) |
| 6-10 mm | 251/55 | 5.40 (3.27-8.91) | <0.001 |  | 3.55 (1.97-6.40) | <0.001 | 17.97 (13.84-22.74) |
| 11-15 mm | 61/18 | 7.27 (3.73-14.14) | <0.001 |  | 3.98 (1.76-8.96) | <0.001 | 22.78 (14.10-33.60) |
| 16-20 mm | 15/19 | 31.19 (14.15-68.76) | <0.001 |  | 21.10 (7.59-58.68) | <0.001 | 55.88 (37.89-72.81) |
| >20 mm | 9/30 | 82.08 (35.11-191.93) | <0.001 |  | 33.76 (11.84-96.26) | <0.001 | 76.92 (60.67-88.87) |
| Abbreviations: CI, confidence interval; ESECC, Endoscopic Screening for Esophageal Cancer in China; LUL, Lugol-unstained lesion; OR, odds ratio; SDA, severe dysplasia and above. ^†^ The crude ORs were derived from univariate logistic regression models. ^‡^ The adjusted ORs were derived from the multivariable logistic regression adjusted by age, gender, body mass index, family history of esophageal squamous cell carcinoma, alcohol consumption, cigarette smoking, eating rapidly, ingestion of leftovers, sharp border of LUL, irregularity, mosaic staining, dark staining border and number of LULs among participants with no dysplasia lesion at baseline. ^§^ The cumulative incidences of SDA of a median of 7 years were calculated as the total of outcome events identified at baseline screening or via follow-up, divided by the number of participants at enrollment in each subgroup. 95% CI was derived from the binomial distribution. ^¶^ Size was defined as the diameter or length, whichever was smaller, of a given LUL. | | | | | | | |

| **Supplementary Table 3. Cumulative risk of having prevalent SDA and incident SDA among 1073 participants stratified by size of LULs during a median of 7-year follow-up** | | | | |
| --- | --- | --- | --- | --- |
|  |  |  |  |  |
| Variables | No. of participants at baseline /No. of prevalent SDA cases | Detection rate of prevalent SDA & 95% CI  (per 100 persons) ^‡^ | No. of non-SDA cases at baseline /No. of incident SDA cases | Cumulative incidence of incident SDA & 95% CI  (per 100 persons) ^§^ |
| Size of LULs ^†^ |  |  |  |  |
| Small (≤5 mm) | 615/14 | 2.28 (1.25-3.79) | 601/10 | 1.66 (0.80-3.04) |
| Medium (6-15 mm) | 385/46 | 11.95 (8.88-15.61) | 339/27 | 7.96 (5.31-11.38) |
| Large (16-20 mm) | 34/14 | 41.18 (24.65-59.30) | 20/5 | 25.00 (8.66-49.10) |
| Extra-large (>20 mm) | 39/24 | 61.54 (44.62-76.64) | 15/6 | 40.00 (16.34-67.71) |
| Abbreviations: CI, confidence interval; LUL, Lugol-unstained lesion; SDA, severe dysplasia and above. ^†^ Size was defined as the diameter or length, whichever was smaller, of a given LUL. ^‡^ Prevalent cases of SDA were defined as those identified at the baseline screening or within 1 year after the baseline screening.  ^§^Incident cases of SDA were defined as those diagnosed both at re-examination and active or passive follow-up. The cumulative incidences of SDA of a median of 7-year were calculated as the total of outcome events identified at baseline screening or via follow-up, divided by the number of participants at enrollment in each subgroup. 95% CI was derived from the binomial distribution. | | | | |
|  |  |  |  |  |
|  |  |  |  |  |
|  |  |  |  |  |
|  |  |  |  |  |
